# Positive Effect of *Cannabis sativa* L. Herb Extracts on Skin Cells and Assessment of Cannabinoid-Based Hydrogels Properties

**DOI:** 10.3390/molecules26040802

**Published:** 2021-02-04

**Authors:** Martyna Zagórska-Dziok, Tomasz Bujak, Aleksandra Ziemlewska, Zofia Nizioł-Łukaszewska

**Affiliations:** Department of Technology of Cosmetic and Pharmaceutical Products, Medical College, University of Information Technology and Management in Rzeszow, Kielnarowa 386a, 36-020 Tyczyn, Poland; tbujak@wsiz.rzeszow.pl (T.B.); aziemlewska@wsiz.rzeszow.pl (A.Z.); zniziol@wsiz.rzeszow.pl (Z.N.-Ł.)

**Keywords:** *Cannabis sativa* L., antioxidants, metalloproteinase inhibitors, cytotoxicity, skin cells, hydrogel

## Abstract

The skin is an organ that is constantly exposed to many external factors that can affect its structure and function. Due to the presence of different cannabinoid receptors on many types of skin cells, cannabinoids can interact directly with them. Therefore, as part of this work, the impact of two types of *Cannabis sativa* L. herb extracts on keratinocytes and fibroblasts was assessed. The content of biologically active compounds such as phenols, flavonoids, chlorophylls and cannabinoids was evaluated. The antioxidant capacity of prepared extracts using the DPPH radical, H_2_DCFDA probe and measurement of superoxide dismutase activity was also assessed. The cytotoxicity of hemp extracts was determined using the Alamar Blue, Neutral Red and LDH assays. The ability of the extracts to inhibit the activity of matrix metalloproteinases, collagenase and elastase, was assessed. Preparations of model hydrogels were also prepared and their effect on transepidermal water loss and skin hydration was measured. The obtained results indicate that hemp extracts can be a valuable source of biologically active substances that reduce oxidative stress, inhibit skin aging processes and positively affect the viability of skin cells. The analysis also showed that hydrogels based on cannabis extracts have a positive effect on skin hydration.

## 1. Introduction

Plant cells produce numerous chemicals that are secreted as physiological components or as by-products of metabolism. They show anti-inflammatory, cytotoxic, but also antibacterial or antifungal properties [1,2,3]. A very large group of them have strong antioxidant properties [4]. To a large extent, their properties can be associated with the regulatory effect of plant substances on the oxidative-reduction processes occurring in cells, including maintaining redox balance [5,6]. Many factors can disturb the oxidative balance, leading to oxidative stress, as a result of which the amount of substances capable of neutralizing free radicals is much lower than the amount of free radicals. Therefore, it is very important to support the body’s endogenous protective system by exogenous antioxidants, which may have a significant impact on the function of various types of important proteins, cell signaling process or a number of enzyme systems [6,7,8].

The variety of chemical compounds found in plant material has been inspiring scientists for years and contributes to the fact that active compounds contained in plant materials are used in many industries [9]. Plant extracts are increasingly used in pharmaceutical and cosmetic research, which aims to create new drugs, supplements and cosmetic products [10]. Modern research methods allow standardization of extracts composition and full quality control of products. Their widespread use in many industries results from their multifunctionality. In addition to antioxidant activity, plant extracts contain a number of active substances that have protective, regenerative and anti-inflammatory properties [7,11,12]. Particularly noteworthy is the group of compounds called cannabinoids occurring naturally in plants as phytocannabinoids and in animal organisms as endocannabinoids. Cannabinoids are lipophilic compounds interacting with cannabinoid receptors present in mammalian cells. Hemp is one of the main sources of phytocannabinoids [13,14] and belongs to the group of phenolic terpenoids and are synthesized primarily in the secretory gland of female flowers. Currently, more than 100 different phytocannabinoids are known [15] and the most important include delta-9-tetrahydrocannabinol (THC), cannabidiol (CBD), cannabidiol acid (CBDA), cannabinol (CBN) and cannabigerol (CBG). One of the most common cannabinoids found in different varieties of cannabis plants is THC. The content of THC in plants ranges from less than 0.2% (in fibrous varieties) up to even 30% in female flowers in other varieties of cannabis [15,16,17]. The phytocannabinoids also include CBD, which, unlike THC, does not activate G-protein-related endocannabinoid receptors, causing no psychostimulatory effect of this compound. In addition, CBD may enhance the beneficial effects of THC by increasing its therapeutic range. It has also been shown that the polyphenolic nature of CBD makes it a powerful antioxidant [18]. It should be noted that in fresh plant material, 95% of CBD is in the form of cannabidiolic acid (CBDA), which has a strong anti-inflammatory effect. Cannabinol (CBN) and cannabigerol (CBG) also exhibit this effect [19,20,21].

In addition, hemp is a rich source of many active substances. A large group of compounds are terpenes and sesquiterpene which are responsible for the characteristic smell of cannabis. In addition, terpene components of hemp may have synergistic properties with phytocannabinoids and enhance their health-promoting effects [22,23]. Polyphenolic compounds, which include flavonoids (flavones and flavonols), stilbenes and lignans, are largely responsible for the antioxidant activity of the plant material. Thanks to the lignin content, hemp raw materials also provide effective protection against UV radiation [19,20].

The aim of this work was to present the hemp extract as a multifunctional ingredient in cosmetic and pharmaceutical preparations intended for skin care. For this purpose, the antioxidant and cytotoxic properties of water-ethanol extracts from *Cannabis sativa* L. were assessed. Due to the fact that oxidative stress significantly affects the condition of the skin, this work includes an assessment of the ability of hemp to scavenge exogenous free radicals, affect the intracellular level of reactive oxygen species and increase the activity of antioxidant enzyme-superoxide dismutase. The work also contains the determination of the content of biologically active compounds such as phenols, flavonoids and cannabinoids. Various cytotoxicity assays have also been carried out, such as Alamar Blue, Neutral Red and LDH to assess the cytotoxicity of the obtained extracts for skin cells-keratinocytes and fibroblasts. In addition, to determine potential anti-aging properties, the ability to inhibit collagenase and elastase activity as well as the effect of hydrogels based on cannabis extracts on transdermal water loss and skin hydration were determined.

## 2. Results and Discussion

### 2.1. Determination of Biologically Active Compounds

Polyphenols and flavonoids are the basic active ingredients of plant extracts. They are responsible for their antioxidant activity, by neutralizing free radicals that may generate oxidative stress. Oxidative stress is one of factors inducing skin aging processes and inhibiting its regeneration ability [7]. Long-term oxidative stress in skin cells (occurring e.g., after prolonged skin exposure to the sun, as well as due to many external factors, e.g., smog) may leads to DNA, protein and lipid damage, disrupting many natural processes, including degradation and synthesis of collagen and elastin-basic proteins that play the most important role in a skin aging process, regeneration or wound healing [24,25,26]. Chlorophyll may also enhance the antioxidant effect [6,24]. The research showed that the extraction method of the hemp herb has significant impact on the concentration of flavonoids and polyphenols, as well as chlorophyll in the obtained extract [15,25]. The results (calculated per 1 g of dry extract and 1 g of dry hemp herb) are presented in Table 1. The extract obtained by ultrasonic extraction is characterized by a higher content of polyphenols, flavonoids and chlorophyll compared to the extract obtained using the traditional method. For the ultrasound (UAE) extract, 20% higher concentration of polyphenols, about 30% higher of flavonoid and twice higher chlorophyll concentration were obtained (Table 1).

Concentration of active ingredients in hemp extract depends on the extraction method, temperature, a type of solvent, as well as the part and variety of the plant from which they are obtained. As shown in previous studies [27,28,29,30,31,32], the total content of phenols per 1 g of dried plant or dry extract is about 0.09–0.56 mg in hemp leaves, 4.7–8.1 mg in flowers, 0.77–51.6 mg in seeds and 10.51–52.58 mg in inflorescences. In the research of Maqsood et al. [26] the influence of different solvents on the concentration of antioxidants in cannabis leaf extracts was compared. It was shown that the highest content of phenols was characterized in aqueous and methanolic extracts, while extracts obtained using organic solvents did not show the content of phenols. The highest content of flavonoids (about 55–60 mg/g of the extract) was found in methanolic and ethanolic extracts. Lower concentrations of these substances were found in chloroform and acetone extracts (about 20 mg/g of the extract). Flavonoids were not found in root extract while inflorescences were characterized by a content of flavonoids about three times lower than the leaves [29]. The lower concentration of flavonoids in extracts analyzed in this work is due to the fact that herb of hemp was used, containing leaves, inflorescences and stems of hemp.

As part of this work, a chromatographic assessment of the amount of individual cannabinoids in the prepared extracts was also carried out. The analysis confirmed that the selection of ultrasonic extraction results in a higher concentration of cannabinoids in *Cannabis sativa* L. extracts compared to the extractions using a traditional method using a magnetic stirrer. The results of the tests showed that the cannabinoids that occur in the largest amount in the obtained extracts are Cannabidiol Acid (CBD-A) and Cannabidiol (CBD) (Table 2).

### 2.2. Assessment of Antioxidant Activity

The antioxidant properties of plants are extremely important in the context of protecting cells against the adverse effects of various external factors. Therefore, the next stage of the study was to assess the prepared UAE and MAE extracts from hemp in terms of their ability to scavenge the DPPH free radical, changes the activity of superoxide dismutase (SOD) and the impact on the amount of reactive oxygen species produced inside the tested cells-fibroblasts and keratinocytes.

**Figure 1:**
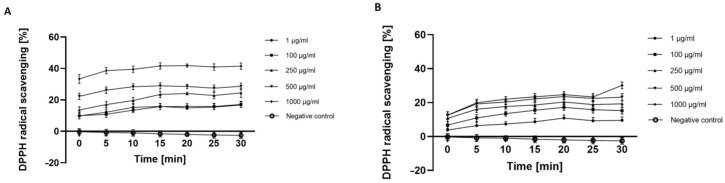
Kinetics of the absorbance changes in DPPH• solutions in the presence of various concentrations (1–1000 µg/mL) of UAE (**A**) and MAE (**B**) extracts of *Cannabis sativa* L. herb. Values are mean of three replicate determinations (*n* = 3).

Analysis aimed at assessing the antioxidant properties of the analyzed extracts by assessing the ability to scavenge the 1,1-diphenyl-2-picrylhydrazyl (DPPH) radical clearly indicated that the extracts of *Cannabis sativa* L. have the ability to neutralize the DPPH radical in a dose-dependent manner. UAE extracts, in which analysis showed a greater amount of biologically active compounds compared to MAE (Table 1 and Table 2), showed better antioxidant properties compared to MAE extracts at the concentration of 500 and 1000 μg/mL. After using the UAE extract, more than 40% free radical scavenging was observed, while in the case of the extract obtained on a magnetic stirrer, this inhibition reached up to 30% for the highest concentration used (1000 μg/mL). In the analysis, it was observed that as the concentration of both extracts increased (in the range of analyzed concentrations), the antioxidant capacity of the extracts was also higher (Figure 1A,B). This correlates with the results obtained by other authors who pointed to the antioxidant properties of chemical compounds whose presence in the analyzed hemp extracts was confirmed in this work [33,34].

**Figure 2:**
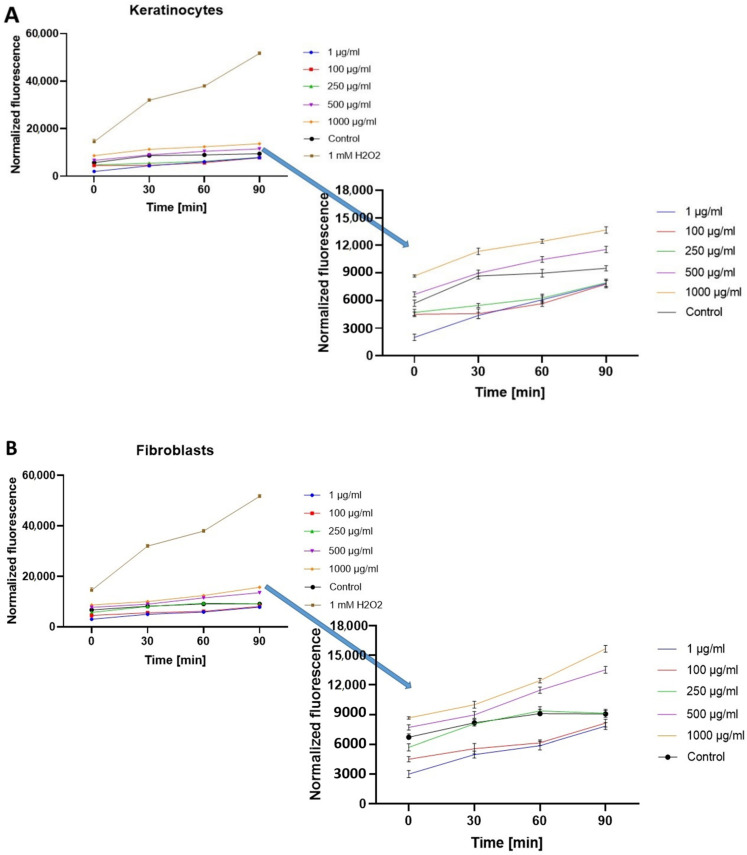
The effect of UAE *Cannabis sativa* L. extract on the 2′,7′-dichlorofluorescein (DCF) fluorescence in HaCaT (**A**) and fibroblasts cells (**B**). The data are expressed as the mean  ±  SD of 3 independent experiments, each of which consisted of 3 replicates per treatment group.

Before starting the analysis using 2′,7′-dichlorodihydrofluorescein diacetate (H_2_DCFDA), it was checked whether the cannabis extracts alone (without the cells tested) affect H_2_DCFDA fluorescence. After excluding these interactions, the plant extracts were tested on cell lines. The research showed that the hemp extract effect varies depending on the concentration used and the cell type. In the case of fibroblasts (BJ), after using UAE extract from *Cannabis sativa* L. in the concentration range of 1–250 µg/mL, the level of intracellular reactive oxygen species (ROS) was below the value obtained for the control (cells not treated with the extract). The cells also treated with 1 mM hydrogen peroxide (H_2_O_2_) were used as a positive control. Compared to the control, doses above 250 μg/mL resulted in a statistically significant increase in intracellular ROS production. (Figure 2A). Similar results were obtained for keratinocyte cells (HaCaT). The two highest concentrations of hemp extract caused a statistically significant increase in ROS production, while the values of normalized fluorescence for concentrations of 250 µg/mL and lower are below the control value which indicates a reduced amount of reactive oxygen species (Figure 2B). In the case of fibroblasts, a statistically significant decrease in ROS production was observed at the concentrations of 1 and 100 µg/mL, while in the case of keratinocytes at the concentrations from 1–250 µg/mL. The obtained results show that lower concentrations of the tested extracts show a protective effect on the cells tested, thereby reducing oxidative stress inside the cells.

**Figure 3:**
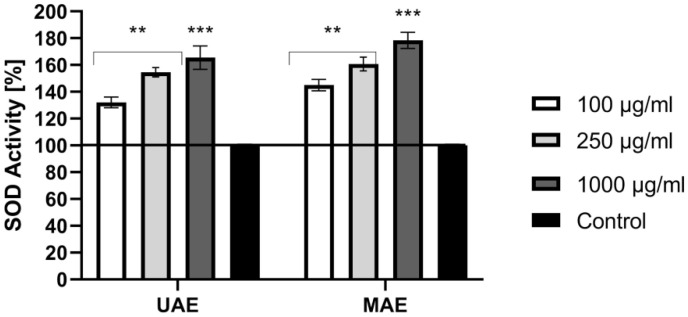
Effect of UAE and MAE extracts from *Cannabis sativa* L. (100, 250, 1000 μg/mL) on superoxide dismutase activity. Data are the mean ± SD of three independent experiments, in which each concentration was tested in duplicate. *** *p* < 0.001, ** *p* < 0.01 versus the control (100%).

In the next step, the ability of the hemp extracts to change the activity of superoxide dismutase (SOD), which is an enzyme that acts as the first stage of antioxidant defense and protects cells from damage by ROS, was assessed [35]. The activity of SOD was measured in cell-free in vitro assay. The conducted analysis indicated that both analyzed extracts show similar ability to increase SOD activity. This effect is dependent on the concentration used and correlates with other analyses performed as part of this work assessing antioxidant capacity (using DPPH and H_2_DCFDA) indicating that as the concentration of the extract increases, the antioxidant properties are stronger (Figure 3). The obtained results indicate that all analyzed concentrations (both UAE and MAE extract) cause statistically significant differences in SOD activity. Superoxide dismutase activity was highest at a concentration of 1000 μg/mL and reached up to 178% and 165% for UAE and MAE extract, respectively. The ability of chemical compounds contained in cannabis extracts to increase SOD activity has also been confirmed by other authors, including in vivo studies [36,37]. Antioxidant abilities of plant extracts, including *Cannabis sativa* L. extract, are extremely important because they allow cells to be protected against oxidative stress, lipid peroxidation or DNA damage. Protection against free radicals is extremely important in the context of skin cells, because these radicals cause damage to the skin’s structure and significantly affect its aging processes [37]. Hence, natural compounds that are capable of scavenging ROS are intensively sought.

### 2.3. Cytotoxicity Assessment

**Figure 4:**
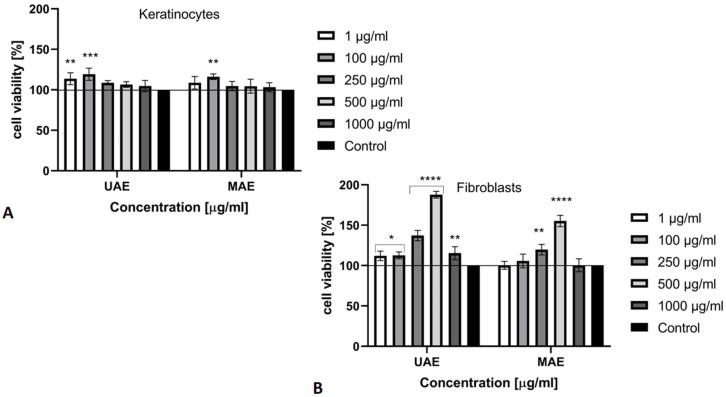
The effect of increasing concentrations of *Cannabis sativa* L. extract (1–1000 μg/mL) on Neutral Red Dye uptake in cultured (**A**) keratinocytes (HaCaT) and (**B**) fibroblasts (BJ) after 24 h of exposure. Data are the mean ± SD of three independent experiments, each of which consists of four replicates per treatment group. For BJ **** *p* < 0.0001, ** *p* = 0.04, * *p* < 0.03 versus the control (100%). For HaCaT *** *p* = 0.004, ** *p* < 0.01 versus the control (100%).

In the next stage of our research, the cytotoxicity of *Cannabis sativa* L. extracts against skin cells (fibroblasts and keratinocytes) was evaluated in vitro using three types of assays (Neutral Red uptake assay, Alamar Blue test and lactate dehydrogenase (LDH) cytotoxicity test). The Neutral Red uptake assay is one of the most used cytotoxicity tests with many biomedical applications [38]. It is based on the ability of viable cells to incorporate and bind the supravital dye neutral red. This weakly cationic dye penetrates cell membranes by nonionic passive diffusion and concentrates in the lysosomes, where it binds by electrostatic hydrophobic bonds to anionic and/or phosphate groups of the lysosomal matrix [39]. Our results obtained with the Neutral Red test have found that *Cannabis sativa* L. extract at all tested concentrations (1–1000 μg/mL) showed no cytotoxic effect on keratinocytes and fibroblasts, and thus does not affect the integrity of cell membranes. Due to the fact that keratinocytes are cells that are directly exposed to various external factors as well as cosmetic preparations applied directly to the skin, it is reasonable to perform cytotoxicity tests on these cells. The highest increase in keratinocytes proliferation, and thus the highest number of active metabolic cells, was observed for extracts at a concentration of 100 μg/mL and this increase reaches even 120% (Figure 4A). What is more, it can be seen that the level of proliferation decreases as the concentration of extracts increases. Due to the possibility of penetration of the ingredients of cosmetic and pharmaceutical preparations through individual skin layers during their topical application, the work examined the cytotoxic effect of obtained hemp extracts on cells that are found in the deeper layers of the skin-fibroblasts. The highest cellular proliferation of fibroblasts was observed at a concentration of 500 μg/mL and this increase reached 188%. The extract obtained by ultrasound-assisted extraction increased the viability of both types of skin cells to a greater extent. The analysis shows that fibroblasts are more sensitive to the effects of *C. sativa* L. extracts compared to keratinocytes (Figure 4B).

**Figure 5:**
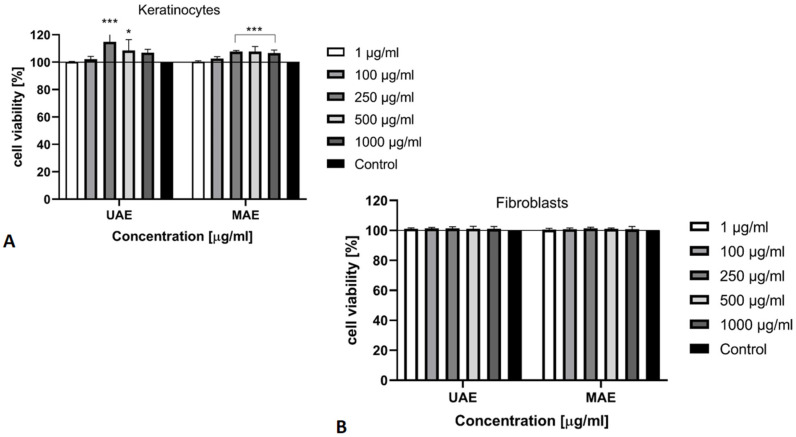
The reduction of resazurin after 24 h exposure to the *Cannabis sativa* L. extract (1–1000 μg/mL) in cultured (**A**) keratinocytes (HaCaT) and (**B**) fibroblasts (BJ). Data are the mean ± SD of three independent experiments, each of which consists of three replicates per treatment group. *** *p* < 0.0005, ** *p* < 0.01, * *p* = 0.0356 versus the control (100%).

The next test used to assess cytotoxicity of *Cannabis sativa* L. extracts was Alamar Blue assay which is a fluorometric method for the detection of metabolic activity of cells. This method is based on the reduction of resazurin (oxidized form 7-hydroxy-3H-phenoxazin-3-1-10-oxide) to resorufin (reduced form), by mitochondrial enzymes that carry diaphorase activity, like NADPH dehydrogenase. Optically, the blue and poorly fluorescent resazurin is gradually transformed by cells into the red, highly fluorescent, resorufin [40,41]. Our results obtained with the Alamar Blue test have also found that analyzed extracts at all tested concentrations (1–1000 μg/mL) showed no cytotoxic effect on two types of cells and thus does not slow down metabolic processes. The cytotoxicity tests performed on keratinocytes showed that the extract at a concentration of 250 μg/mL showed the most favorable effect, where the increase in metabolic activity of the tested cells reaches 115% (Figure 5A). The analysis carried out using the fibroblast cell line demonstrated slight changes in proliferation depending on the concentration of the extracts. The level of cell proliferation of fibroblasts for all concentrations is similar and this only increase reaches 101% (Figure 5B).

**Figure 6:**
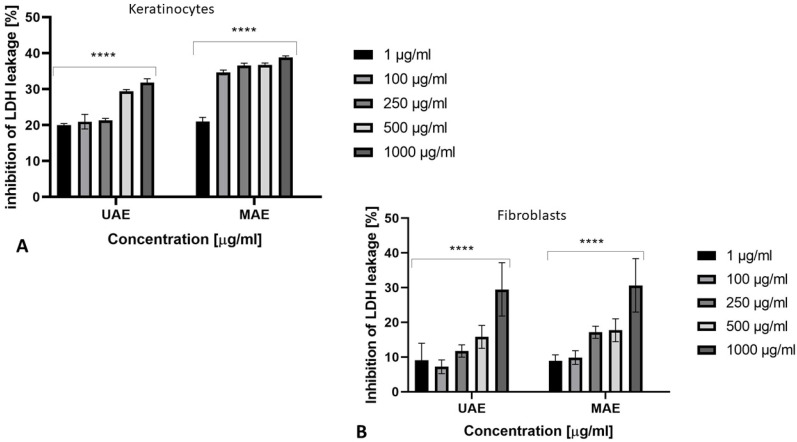
The release of LDH after 24 h exposure to the *Cannabis sativa* L. extract (1–1000 μg/mL) in cultured (**A**) keratinocytes (HaCaT) and (**B**) fibroblasts (BJ). Data are the mean ± SD of three independent experiments, each of which consists of three replicates per treatment group. **** *p* < 0.0001 versus the control (0%).

The cytotoxic effect of the obtained extracts was also assessed using LDH cytotoxicity assay which is a simple, reliable colorimetric method of quantitatively assaying cellular cytotoxicity. The assay can be used with different cell types for assaying cell mediated cytotoxicity as well as cytotoxicity mediated by chemicals and other test compounds. The assay quantitatively measures a stable cytosolic enzyme LDH, which is released upon cell lysis. The released LDH is measured with a coupled enzymatic reaction that results in the conversion of a tetrazolium salt (INT) into a red color formazan. The LDH activity is determined as NADH oxidation or INT reduction over a defined time period [42,43]. At each concentration, there was no significant membrane damage (LDH release). The cell viability results indicate that *C. sativa* L. extracts are nontoxic to keratinocytes and fibroblasts. There were also no changes in extracellular LDH levels after exposure to *C. sativa* L. extract. These values are given as the percentage of the negative control (untreated with tested extracts). A correlation between extract concentration and LDH release was also observed in both analyzed skin cells. With decreasing concentration of extract, membrane damage was less noticeable. In summary, it should be noted that all cytotoxicity tests performed indicate that the extract of *Cannabis sativa* L. does not show toxic effects on the cell lines tested (Figure 6A,B).

The results obtained in these studies indicate the lack of cytotoxicity of *Cannabis sativa* L. extracts to skin cells, especially fibroblasts, which may suggest their potential use as biologically active compounds in the pharmacological, dermatological and cosmetic industries. As mentioned earlier, *C. sativa* L. extracts can be seen as extremely valuable ingredients not only in food products, but also in cosmetic preparations or dietary supplements due to their good protective effect on our body. Studies on keratinocytes and fibroblasts have confirmed that the cannabinoids present in this plant exert anti-inflammatory and protective effects [44]. The skin cells like keratinocytes and fibroblasts are involved in wound healing with the other skin cells [45]. Keratinocytes function are regulated by a variety of cytokines, growth factors and chemokines and in turn, these cells release a few of proinflammatory mediators including interleukin-1 beta (IL-1β), IL-6, IL-8, tumor necrosis factor alpha (TNFα), and transforming growth factor alpha and beta, as well as vascular endothelial growth factor (VEGF), a potent mitogen for endothelial cells, playing a pivotal role in angiogenesis and psoriasis [46,47]. Many authors have shown that cannabinoids present in *Cannabis sativa* L. can have protective effects on the skin. Δ9-THC, CBN and CBD were shown to inhibit keratinocyte proliferation in the low micromolar range and in a cannabinoid receptor independent manner [48]. Sangiovanni et al. have shown that *C. sativa* L. extract is able to inhibit the release of mediators of inflammation involved in wound healing and inflammatory processes occurring in the skin. What is more, UV radiation, especially UVB, also decreased endocannabinoids: anandamide (AEA) and 2-arachidonoylglycerol (2-AG), and significantly increased palmitoylethanolamide (PEA) levels. These changes were significantly greater in keratinocytes from psoriatic patients compared to healthy individuals. CBD counteracted both the reduction of AEA in keratinocytes from healthy individuals, as well as the increase in the level of PEA in psoriatic keratinocytes (both with and without UV irradiation) [49,50]. Thus, CBD can also indirectly modify the activation of CB1 and CB2 receptors through AEA and vanilloid receptor (TRPV1) [51]. Published literature data indicate that PEA may reduce the expression and the levels of inflammatory cytokines in skin diseases through this mechanism [52]. Regarding the effect of cannabinoids on fibroblasts, there are still few publications on this subject. Various studies do not show the cytotoxic effects of cannabinoids on skin cells, but there is still little evidence to show a significant cell proliferation of fibroblasts so it needs to extend research [53,54].

### 2.4. Assessment of Matrix Metalloproteinases Inhibition

**Figure 7:**
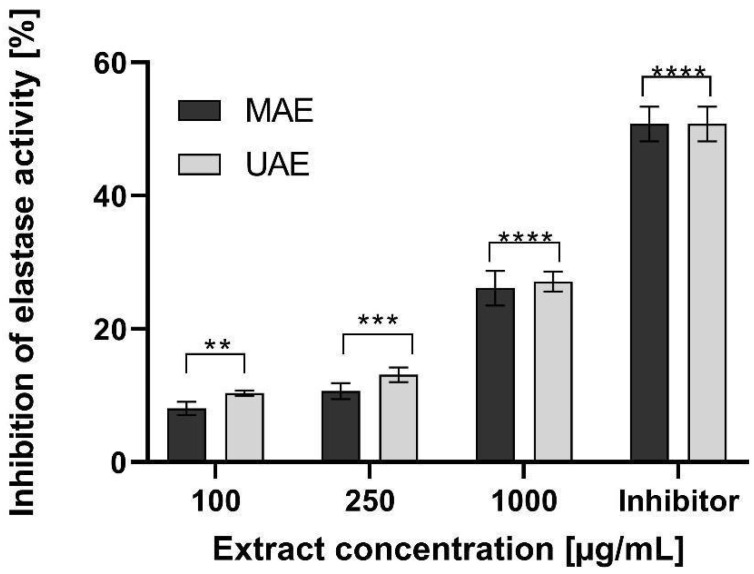
Influence of hemp extracts on elastase inhibition. The inhibitor used was Methyl-4-[[(2*S*) -1-[[(2*S*)-1-[(2*S*) -2-[[(3*S*)-1-chloro-4-methyl-2-oxopentane -3-yl] carbamoyl] pyrrolidin-1-yl]-1-oxopropan-2-yl] amino] -1-oxopropan-2-yl] amino] -4-oxobutanoate. Data are the mean ± SD of three independent experiments, each of which consists of three replicates per treatment group. ** *p* < 0.0012, *** *p* < 0.0004, **** *p* < 0.0001 versus the control.

**Figure 8:**
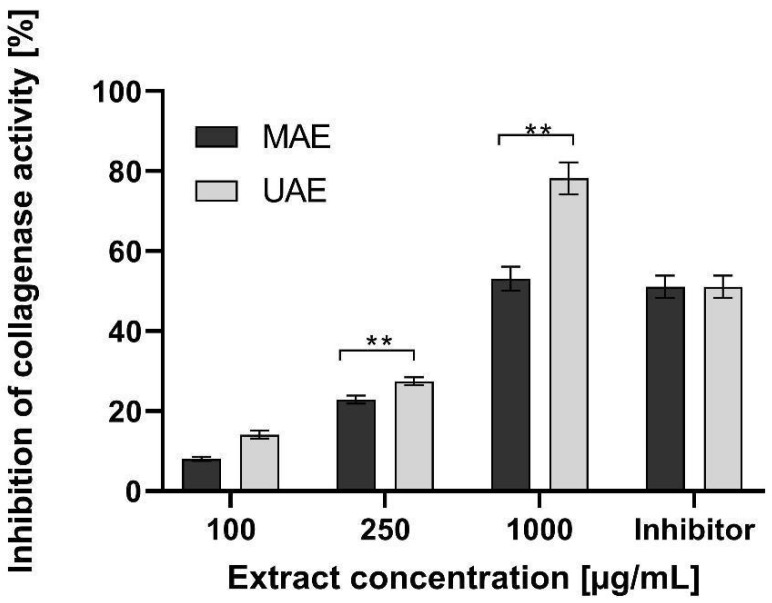
Influence of hemp extracts on collagenase inhibition. Data are the mean ± SD of three independent experiments, each of which consists of three replicates per treatment group. ** *p* < 0.001 versus the control.

Elastin and collagen are the major skin building proteins. They are responsible for an adequate strength, flexibility, elasticity as well as hydration of the skin. These proteins play an important role not only in the skin aging process, but also in wound healing and skin regeneration. Elastase and collagenase enzymes are responsible for a degradation of elastin and collagen structure in the skin. Their activity may be induced by free radicals and UV radiation, as well as internal and genetic conditions [55]. In these studies, the effect of hemp extracts on elastase and collagenase enzymes activity was performed. Results are shown in Figure 7 and Figure 8.

It was found that hemp extracts decreased activity of elastase and collagenase and have the ability to inhibit these enzymes. In the case of elastase activity, addition of increased concentration of hemp extracts resulted in an increase in elastase inhibition. Extract concentration of 100 µg/mL was characterized by a low ability to elastase inhibition. The results for these extracts were about 10%. At extract concentration of 1000 µg/mL, the ability to elastase inhibition increased significantly up to 30%. It was not observed the significant influence of an extraction method on the analysed parameter (Figure 7). The extraction method had a significant influence on the collagenase inhibition. Activity of this enzyme also depended on the extract concentration. The ultrasound extract had much stronger properties to collagenase inhibition. At a concentration of 250 µg/mL, the MAE and UAE extract showed the ability to reduce collagenase activity at the level of about 25% and 30%, respectively. As concentrations rise, the increase of collagenase inhibition by extracts was observed. At 1000 µg/mL, the value of the analyzed parameter was about 55% for the MAE extract. For UAE extract, collagenase inhibition was significantly stronger and was about 80% (Figure 8). The effect of cannabis extracts on the activity of collagenase and elastase enzymes has not yet been studied. However numerous literature data, based on clinical and animal studies, show the significant influence of cannabinoids (e.g., THC, CBD) derived from hemp in wound and burns healing process, skin regeneration, delays of skin ageing and relieving of pruritus or pain. Cannabinoids have also strong anti-inflammatory and antibacterial properties [55,56,57,58]. In addition to supporting wound healing, cannabinoids may be an effective ingredient in the treatment of dermatoses, psoriasis, atopy, skin allergies and skin melanoma [55,56,57]. This effect is attributed to an endocannabinoid system (ECS) formed by endocannabinoids and their CB1 and CB2 receptors. Recent studies showed that CB1 and CB2 receptors have endogenous ligands located in the skin. It may indicate that the skin has its own ECS system, which is responsible for skin health by maintaining a skin homeostasis. Cannabinoids can act as a stimulant or an inhibitory agent for ECS, e.g., by inhibiting or promoting proliferation of keratinocytes, sebum production or inhibition of inflammatory promoters [56,57,58,59]. The regenerating effect of cannabinoids and their positive effect on the skin condition may therefore be a result of ECS action and ability of cannabinoids to inhibit activity of collagenase and elastase.

### 2.5. Assessment of Hydrogel Properties

**Figure 9:**
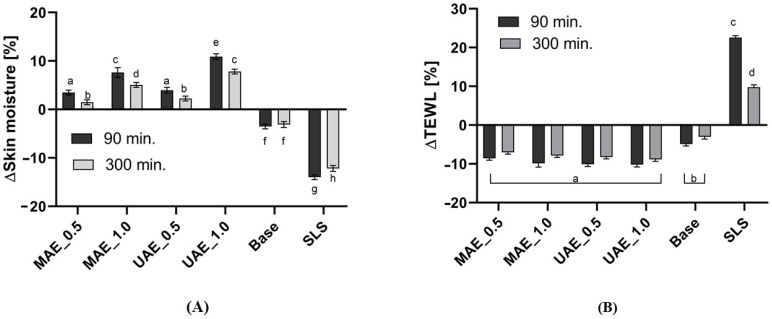
Influence of hemp extracts on skin hydration (**A**) and TEWL (**B**). Different letters on the charts indicate significant differences between groups (*p* < 0.05). The determinations were made in 5 replicates. MAE_0.5 and MAE_1.0 are hydrogels containing 0.5 and 1.0% of the dry extract obtained using traditional method and UAE_0.5 and UAE_1.0 are hydrogels containing 0.5 and 1.0% of the dry extract obtained by ultrasound-assisted extraction.

In the case of many dermatological problems like atopy, dermatosis, psoriasis, as well as in the process of wound healing, it is extremely important to keep an adequate level of skin moisture. Due to many external factors, drinking too small amounts of water, dietary, and above all, cleansing cosmetics used every day, the level of skin moisture may decrease. Cleansing cosmetics may also disturb the hydrolipid skin barrier, which can increase the amount of water that evaporates from the skin. It may impede and slow down processes of wounds, scars and burns healing [54]. Therefore, the influence of hemp extracts (in the form of hydrogels) on skin moisture and transepidermal water loss (TEWL) was evaluated. Before application of hydrogels the forearm skin was washed with 1% SLS solution. SLS is one of the most popular cleaning agents used in formulations of cleansing cosmetics and it has a strong ability to dry the skin and damage the skin’s lipid barrier [55,56,57,58,59,60]. The results of the corneometric and TEWL study are shown in Figure 9. In these studies, a hydrogel based on hydroxyethylcellulose (base sample) and the same hydrogel with addition of analysed hemp extracts was applied on the skin of 15 volunteers. 90 and 300 min after the hydrogels application, changes in skin moisture and TEWL were measured.

Application of hemp extracts in the hydrogel form have a positive effect on the skin condition. After the skin cleaning process with 1% of SLS level of the skin moisture was decreased. In related to a control field without treatment with any samples, after application of SLS solution it was noted decrease in the skin hydration by about 14% and 11% (after 1.5 h and 5 h, respectively). The negative value of a skin moisture change means a strong ability of this surfactant to dry the skin. Analogically, SLS also had a strong ability to disturb the skin hydrolipid balance as evidenced by the positive value of TEWL change 1.5 and 5 h after SLS application. 5 h after SLS application it was observed that skin did not return to its physiological state and the moisture level was lower and the TEWL value was higher than values observed for the control field. Drying effect and damaging of skin barrier by surfactants can make it difficult to wound healing and skin regeneration, as well as exacerbate symptoms of atopy, psoriasis and sensitive skin and induce skin irritations. After application of hydrogels containing hemp extracts on sodium lauryl sulfate (SLS)-treated skin, the skin condition improved significantly. Hemp hydrogels eliminated adverse effect of analyzed surfactant on the skin moisture and TEWL. The use of hydrogels without extracts (base sample) also improved the skin condition, but the effect of base sample was not so strong. Hydrogels containing 0.5% of extracts acted as skin moisturizer. 5h after application of these hydrogels, the skin returned to a physiological state, and the change in skin moisture was close to the value observed for the control field. For MAE_0.5 i UAE_0.5 hydrogels it was not observed a significant influence of hemp extraction method on the skin moisture (*p* < 0.05). Samples with 1.0% of the extract in the formulation had a stronger skin moisturizing potential and for the UAE extract the long-lasting, moisturizing properties were significantly better than for the MAE extract. After 5 h, the increase in skin moisture (relating to the control field) was about 10% for UAE_1.0 sample and 5% for MAE_1.0 hydrogel. TEWL analysis showed that addition of hemp extracts in the formulation of hydrogels have an influence to restoring of the hydrolipid balance and to rebuilding of the hydrolipid barrier of the skin, which are damaged in a cleaning process by SLS. Analysis of results indicated that the influence of the hemp extraction method and concentration of the extract on hydrogels properties was not significant. Similar TEWL results were obtained for all samples containing the extract in the formulation. Change of TEWL after application of these samples was about 20–30% lower than for SLS treated skin, which indicates the protective effect of hemp hydrogels against water loss from the epidermis. Plant extracts are a source of many substances that moisturize the skin. These are mainly substances with hydroxyl groups in their molecules that can form a hydrogen bond with water, thus binding water in the epidermis. The active components of plant extracts are mainly proteins, carbohydrates, as well as polyphenols and flavonoids, containing several hydroxyl groups in the molecule [55]. Hemp extracts are rich in this kind of substances and they are responsible for the moisturizing of the skin [25]. Proteins and carbohydrates due to their high molecular weight may act as an occlusive film on the skin surface causing decrease of transepidermal water loss. Cannabinoids because of their hydrophobicity and a low molecular weight may act on the skin surface as an occlusive film and also penetrate into deeper layers of the epidermis, retaining water and providing a long-lasting moisturizing effect [55].

## 3. Materials and Methods

### 3.1. Plant Material and Extraction Procedure

Plant material was purchased from a local herbal store. *Cannabis sativa* L. herb were collected on controlled and ecological plantations. No chemical fertilizers nor plant protection products were used for the cultivation. As part of this work, two types of hemp extracts were prepared using ultrasound assisted extraction (UAE) and magnetic stirrer assisted extraction (MAE). The extract was prepared by extracting 15 g of *Cannabis sativa* L. herb in a 100 g water-ethanol solution (20:80). UAE was obtained according to the method described by Yang et al. in an ultrasonic bath (Digital Ultrasonic Cleaner, Berlin Germany) equipped with a time controller [61]. The mixtures were extracted at room temperature for 60 min (6 cycles of 10 min). When the extract temperature reached 25 °C the extract was rapidly cooled in the ice to 22–23 °C. MAE extracts were made on a magnetic stirrer at 300 rpm (RCT basic, IKA, Staufen, Germany). Extraction was carried out for 60 min. The obtained extracts were then collected and filtered three times through Whatman No. 1 filter paper using vacuum filtration. After filtration, the UAE and MAE extracts were evaporated under reduced pressure at 40 °C. A stock solution at the concentration of 100 mg/mL was prepared from the dried extracts, and was stored in the dark at 4 °C until further analysis.

### 3.2. Determination of Biologically Active Compounds

#### 3.2.1. Total Phenolic Content Determination

The concentration of total phenolic compounds in *Cannabis sativa* L. extracts was determined spectrophotometrically using the Folin-Ciocalteu method described by Singleton et al. with some modifications [62]. Gallic acid (GA) was used as standard. For this purpose, 300 μL of hemp extract sample at various concentrations was mixed with 1500 μL of Folin Ciocalteu 1:10 reagent. After 6 min of incubation, 1200 μL of a 7.5% sodium carbonate solution was added to the analyzed samples. Samples were mixed and incubated in the dark at room temperature (about 22 °C) for 2 h. Absorbance was read at λ = 740 nm on an Aquamate Helion spectrophotometer (Thermo Scientific, Waltham, MA, USA). To calculate the total concentration of phenols in hemp extracts (both UAE and MAE), a gallic acid (GA) calibration curve (in the 10–100 mg/mL concentration range) was used. As a negative control was used ethyl alcohol. The measurements were made in triplicate and the results obtained were averaged.

#### 3.2.2. Total Flavonoids Content Determination

The concentration of flavonoids in the analyzed hemp extracts was measured spectrophotometrically using aluminum nitrate nonahydrate. For this purpose, the method described by Matejić et al. with modifications was used [63]. 2400 μL of the previously prepared reaction mixture consisting of 80% C_2_H_5_OH, 10% Al (NO_3_)_3_ ×9 H_2_O and 1M C_2_H_3_KO_2_ were mixed with 600 μL of the tested sample of the extract at various concentrations. After 40 min incubation at room temperature (about 22 °C) in the dark, the absorbance of the prepared mixtures at λ = 415 nm was measured using a FilterMax F5 AquamateHelion spectrophotometer (Thermo Scientific). The total flavonoid concentration in the analyzed samples was calculated from the calibration curve for quercetin (Qu) hydrate (in the concentration range of 10–100 mg/mL). Measurements were made in triplicate for each sample.

#### 3.2.3. Determination of Chlorophyll Content

The chlorophyll content of hemp extracts was determined by spectrophotometry. Stock solution of dry MAE and UAE extracts at a concentration of 100 µL/mL in 80% acetone was prepared. The absorbance of the solutions was measured at λ = 645 nm and λ = 663 nm using UV-Vis spectrophotometer Filter Max 5 (Thermo Scientific). The results were expressed as the content of chlorophyll a and b and the total content of chlorophyll (a + b), calculated on 1 g of dry hemp extract (DWE) and hemp herb (DWH). The final result is the average of three independent determinations.

#### 3.2.4. Determination of Cannabinoids

The test was performed in an external accredited laboratory. A Hewlett Packard HPLC system (DionexUltiMate 3000 RS, Thermo Fisher Scientifilic, Sunnyvale, CA, USA) equipped with an integrator and a UV/VIS detector as well as a C_18_ column filled with silica gel was used in the tests. The study was conducted in a reverse phase system. The mobile phase was mixing methanol and phosphoric acid (75:25). Analysis conditions: 1 mL/min, 35 °C, detection at λ = 230 nm. Cannabinoid concentration was read from the standard curve using external standards.

### 3.3. Assessment of Antioxidant Activity

#### 3.3.1. DPPH Radical Scavenging Assay

The ability of the extracts obtained to scavenge free radicals was determined using the stable DPPH radical. For this purpose, the methodology described by Brand-Williams et al. has been applied [64]. Briefly, 33 μL of cannabis extracts tested at various concentrations (0.1–10%) were mixed with 167 μL methanol solution of DPPH (4 mM) and transferred to a 96 well plate. The analyzed samples were mixed thoroughly by shaking. In the next step, the absorbance of the samples at λ = 517 nm was measured. Measurements were made every 5 min for 30 min on a UV-ViS Filter Max 5 spectrophotometer (Thermo Scientific). Three independent replicates were performed for each concentration. Water-ethanol solution (20:80) with a DPPH solution was used as a control. The antioxidant capacity of *Cannabis sativa* L. extracts was expressed as a percentage of DPPH inhibition using the following equation:

$$\%\;\mathrm{DPPH}\;\mathrm{scavenging}=\frac{\mathrm{Abs}\;\mathrm{control}\;-\;\mathrm{Abs}\;\mathrm{sample}}{\mathrm{Abs}\;\mathrm{control}}\;\times \;100\%$$

where Abs control is the absorbance of the control sample (containing DPPH and water-ethanol solution), Abs sample is the absorbance of the test sample (containing DPPH and test extract).

#### 3.3.2. Detection of Intracellular Levels of Reactive Oxygen Species (ROS)

In order to determine the ability of the analyzed UAE and MAE hemp extracts to generate the intracellular production of reactive oxygen species in HaCaT and fibroblast cells, a fluorogenic H_2_DCFDA dye was used. After passive diffusion of this compound into the cells, it is deacetylated by intracellular esterases to a non-fluorescent compound. In the presence of reactive oxygen species it is oxidized and transformed into highly fluorescent DCF [65].

To determine the intracellular level of ROS in HaCaTs and fibroblasts, cells were seeded in 96 well plates at a density of 1 × 10^4^ cells per well. Then, cells were cultured in an incubator for 24 h. DMEM medium was removed and replaced with 10 μM H_2_DCFDA (Sigma Aldrich, Sant Louis, MO, USA) dissolved in serum free DMEM medium. HaCaT and BJ cells were incubated in H_2_DCFDA for 45 min and then incubated with *Cannabis sativa* L. extracts in the concentration range of 1–1000 μg/mL. Cells treated with 1 mM hydrogen peroxide (H_2_O_2_) were used as positive controls. The control samples were cells untreated with the tested extracts. DCF fluorescence was measured every 30 min for 90 min using a FilterMax F5 microplate reader (Thermo Fisher Scientific) at a maximum excitation of 485 nm and emission spectra of 530 nm.

#### 3.3.3. Determination of Superoxide Dismutase (SOD) Activity

Colorimetric Superoxide Dismutase Activity Assay kit (ab65354, Abcam, Cambridge, UK) was used to determine the impact of the cannabis extracts on the activity of the antioxidant enzyme involved in the defense system against reactive oxygen species. MAE and UAE *Cannabis sativa* L. extracts in concentrations of 100, 250 and 1000 µg/mL were used for the analysis. Recombinant human Superoxide Dismutase 1 protein (ab112193, Abcam) was used to prepare the standard curve. Samples were prepared in 96-well plates (clear bottoms) and the analysis was performed according to the manufacturer’s instructions. Initially, 200 μL of WST working solution was added to each well. Then, test samples were prepared by adding 20 µL Enzyme Working Solution and 20 µL of hemp extracts (with a final concentration 100, 250, 1000 µg/mL) to these wells. Three different blank samples were also prepared as recommended. Blank 1 was prepared by adding 20 µL Enzyme Working Solution and 20 µL dd H_2_O to the wells. To blank 2 20 µL Dilution Buffer and 20 µL hemp extracts (with a final concentration 100, 250, 1000 µg/mL) was added. 20 µL of Dilution Buffer and 20 µL dd H_2_O were added to blank 3. All samples were mixed thoroughly by shaking and incubated at 37 °C for 20 min. The absorbance of the samples was then measured at λ = 450 nm using a microplate reader (FilterMax F5, Thermo Fisher). All samples were prepared in duplicate according to the manufacturer’s instructions. The ability to inhibit SOD activity by the analyzed samples was calculated from the equation:

$$\%\;\mathrm{SOD}\;\mathrm{Activity}=\frac{\left(\mathrm{Ablank}1\;-\;\mathrm{Ablank}3\right)\;-\;\left(\mathrm{Asample}\;-\;\mathrm{Ablank}2\right)}{\left(\mathrm{Ablank}1\;-\;\mathrm{Ablank}3\right)}\;\times \;100\;\%$$

### 3.4. Cell Culture

In this work, two skin cell lines were used. HaCaT cells (normal human keratinocytes) were purchased from CLS Cell Lines Service (Eppelheim, Germany), while BJ cells (fibroblasts, ATCC^®^CRL-2522 ™) were obtained from the American Type Culture Collection (Manassas, VA, USA). Both cell lines were maintained in DMEM (Dulbecco’s Modification of Eagle’s Medium, Biological Industries, Cromwell, CO, USA) with L-glutamine, 4.5 g/L glucose and sodium pyruvate. The medium was additionally supplemented with 10% (*v*/*v*) fetal bovine serum (FBS, Gibco, Waltham, MA, USA) and 1% (*v*/*v*) antibiotics (100 U/mL penicillin and 1000 µg/mL streptomycin, Gibco). Cells were kept in an incubator at 37 °C in a humid atmosphere of 95% air and 5% carbon dioxide (CO_2_).

### 3.5. Cell Viability Assay

After the cultured cells (HaCaT and BJ) reached the appropriate confluence, the DMEM culture medium was removed from the culture plate (VWR, Radnor, PE, USA) and the cells were washed twice with sterile PBS (phosphate buffered saline, Gibco). The cell layer was trypsinized using Trypsin/EDTA (Gibco), and then the cells were suspended in a fresh medium. In the next step, cells were plated into 96 well plates (separate plates for both cell types). After the attachment of HaCaT and fibroblasts to the bottom of the plates, the cells were incubated with various concentrations (1, 100, 250, 500 and 1000 μg/mL) of *Cannabis sativa* L. extracts. The cells were cultured in an incubator for 24 h. Controls were cells cultured in DMEM medium without the addition of extracts.

#### 3.5.1. Neutral Red Uptake Assay

The neutral red uptake test (Sigma Aldrich) was used in the studies to assess the viability of skin cells treated with the tested extracts. This test is based on the protocol described by Borenfreund et al. [66]. After exposure to MAE and UAE extracts from *Cannabis sativa* L., cells were incubated for 2 h with a neutral red dye (40 μg/mL) which was dissolved in serum-free medium (DMEM). After incubation, the cells were washed with phosphate buffered saline (PBS) and 150 μL decolorizing buffer (C_2_H_5_OH/CH_3_COOH/H_2_O_2_, 50/1/49%) was added to each well. After shaking the test cells for 15 min, the absorbance of dissolved dye at λ = 540 nm was determined using a FilterMax F5 Multi-Mode microplate reader (Thermo Fisher). The average optical density of the control cells was set to 100% viability and was used to calculate the percentage of viable cells in the experimental samples. The experiments were repeated three times using four wells for each concentration of extracts.

#### 3.5.2. Alamar Blue Assay

To assess the cytotoxicity of the tested extracts and check their effect on cell viability the Alamar Blue assay (R7017, Sigma) was used. This assay is based on the initial protocol described by Page et al. [67]. After exposure of the cells to individual concentrations of the analyzed hemp extracts (1–1000 μg/mL), a solution of resazurin with a final concentration of 60 μM was added to the wells and incubated for 2 h at 37 °C in the dark. Fluorescence was measured at λ = 570 nm using a microplate reader (FilterMax F5, Thermo Fisher). Controls were cells cultured in DMEM medium without the addition of extracts. The experiments were carried out in three independent experiments in which the fluorescence of cells in four wells was measured for each extract concentration. Results are expressed as percent of cell viability compared to control (100%).

#### 3.5.3. Lactate Dehydrogenase (LDH) Cytotoxicity Assay

Another test used to assess cytotoxicity of the tested hemp extracts was a high-throughput, reliable colorimetric method for quantifying cellular cytotoxicity. The activity of LDH in the studied extracts was determined using a commercially available kit (Cytoscan™ LDH Cytotoxicity Assay) from G-Biosciences (A Geno Technology, St. Louis, MO, USA). The assay is based on the conversion of lactate to pyruvate in the presence of LDH with parallel reduction of NAD. The test was carried out according to the instructions provided with the reagents. Analyzes were performed by seeding cells (keratinocytes and fibroblasts) into 96 well plates in DMEM medium. After attachment of the cells to the bottom of the wells, the plates were treated with MAE and UAE extracts at concentrations of 1–1000 μg/mL (Compound Treated). To prepare Spontaneous LDH Activity Control, sterile, ultrapure water was added to the wells instead of the tested extracts. To obtain Maximum LDH Activity Control, according to the instructions, 10 μL of Lysis Buffer was added to the wells. Following exposure the extracts diluted in DMEM, medium was removed and then the culture supernatant was collected and incubated with 50 μL reaction mixture. After incubation at room temperature for 30 min, the reaction was stopped by adding 50 μL Stop Solution. To determine LDH activity, absorbance at λ = 490 nm and λ = 680 nm was measured. Cytotoxicity of the analyzed extracts was calculated using the following equation:

$$\%\;\mathrm{Cytotoxicity}=\frac{\mathrm{Compound}\;\mathrm{Treated}-\mathrm{Spontaneous}\;\mathrm{LDH}\;\mathrm{Activity}}{\mathrm{Maximum}\;\mathrm{LDH}\;\mathrm{release}-\mathrm{Spontaneous}\;\mathrm{LDH}\;\mathrm{Activity}}\;\times \;100\;\%$$

### 3.6. Assessment of Matrix Metalloproteinases Inhibition

#### 3.6.1. Determination of Anti-Collagenase Activity

The ability of the tested *Cannabis sativa* L. extracts to inhibit collagenase activity was analyzed using a fluorometric Collagenase Inhibitor Screening Kit (ab211108, Abcam). UAE and MAE extracts in concentrations of 100, 250 and 1000 µg/mL were used for the analysis. Samples were prepared for analysis in a 96 well plate with clear flat bottom. In the first step, collagenase (COL) was dissolved in Collagenase Assay Buffer (CAB). The test samples were prepared by mixing the analyzed hemp extracts with COL and CAB. Inhibitor control samples were prepared by mixing inhibitor (1,10-Phenanthroline (80 mM)) with diluted collagenase and CAB buffer. Enzyme control wells were prepared by mixing diluted COL with CAB. The CAB buffer was used as background control. The samples were incubated for 15 min at room temperature. In the meantime, a reaction mixture was prepared by mixing the collagenase substrate with CAB. Then, the reaction mixture was added to the prepared samples and mixed thoroughly. The fluorescence was immediately measured with an excitation wavelength of λ = 490 nm and emission λ = 520 nm using a microplate reader (FilterMax F5, Thermo Fisher). The measurement was made in kinetic mode, for 60 min at 37 °C. All samples were prepared in duplicate according to the manufacturer’s instructions. The ability to inhibit COL activity by the analyzed samples was calculated from the equation:

$$\%\;\mathrm{relative}\;\mathrm{COL}\;\mathrm{inhibition}=\frac{\mathrm{enzyme}\;\mathrm{control}-\mathrm{sample}}{\mathrm{enzyme}\;\mathrm{control}}\;\times \;100\%$$

#### 3.6.2. Determination of Anti-Elastase Activity

Fluorometric Neutrophil Elastase Inhibitor Screening Kit (ab118971, Abcam) was used to determine the ability of the extracts to inhibit the activity of the neutrophil elastase (NE) enzyme. MAE and UAE *Cannabis sativa* L. extracts in concentrations of 100, 250 and 1000 µg/mL were used for the analysis. Samples were prepared in 96-well black plates (clear bottoms) for fluorometric assay and the analysis was performed according to the manufacturer’s instructions. Briefly, neutrophil elastase enzyme solutions, NE substrate and inhibitor control (SPCK) were prepared as initially as recommended. The neutrophilic elastase inhibitor used in the analyzes was the chemical compound with the formula C_22_H_35_C_l_N_4_O_7_ (CAS number 65144-34-5). It is a strong, irreversible elastase inhibitor that was part of a kit designed to measure the activity of this enzyme. Then diluted NE solution was added to all wells. Test samples, inhibitor control and enzyme control (Assay Buffer) were applied to subsequent wells. All samples were prepared in duplicate according to the manufacturer’s instructions. The samples were mixed thoroughly on a shaker and the plate incubated at 37 °C for 5 min. The fluorometric reaction mix was then prepared by mixing Assay Buffer and substrate. The prepared reaction mixture was added to each sample and mixed thoroughly. The fluorescence was immediately measured with an excitation wavelength of λ = 400 nm and emission λ = 505 nm using a microplate reader (FilterMax F5, Thermo Fisher). The measurement was made in kinetic mode, for 30 min at 37 °C protected from light. The ability to inhibit NE activity by the analyzed samples was calculated from the equation:

$$\%\;\mathrm{relative}\;\mathrm{NE}\;\mathrm{activity}=\frac{\Delta \mathrm{RFU}\;\mathrm{test}\;\mathrm{inhibitor}}{\Delta \;\mathrm{RFU}\;\mathrm{Enzyme}\;\mathrm{control}}\;\times \;100\%$$

### 3.7. Hydrogel Preparation

The base hydrogel was a 1.2% aqueous solution of hydroxyethyl cellulose (HEC). HEC was added to water and mixed on a mechanical stirrer (Chemland O20, Hamburg, Germany) using a propeller stirrer and stirring speed of 250 rpm. The polymer solution was heated to 60 °C and then cooled to room temperature while stirring to cross-link of HEC. Hydrogels containing hemp extracts were prepared by an analogous method. Hemp extracts were added to HEC hydrogel after cooling. Dry cannabis extracts were added to the hydrogel as a 100 mg/mL solution, using 80% solution of 1,3-propanediol to dissolve them. Four hemp hydrogels were obtained: MAE_0.5 and MAE_1.0, containing 0.5 and 1.0% of the dry extract obtained using traditional method and UAE_0.5 and UAE_1.0, containing 0.5 and 1.0% of the dry extract obtained by ultrasound- assisted extraction.

### 3.8. Transepidermal Water Loss (TEWL) and Skin Hydration Measurements

TEWL and skin hydration measurements were conducted using a TEWAmeter^TM^ 300 probe and Corneometer CM 825 probe connected to MPA adapter (Courage + Khazaka Electronic, Köln, Germany). The study was conducted on 15 volunteers in the age of 28–36. Before the study, each volunteer was informed about the study procedure, research material and contraindications. Each of the volunteers was healthy and signed a declaration of voluntary participation in the study.

Six areas (2 × 2 cm in size) were marked on the forearm skin. 0.2 mL of 1% SLS solution was applied to 5 fields. One field (control field) was not treated with any sample. The SLS solution was gently spread over every skin fragment, and then rinsed with distilled water and dried with a paper towel. After 10 min, 0.2 g of hydrogels were applied on 4 fields of skin treated with SLS. Dry-out hydrogels were removed from the skin with a paper towel 30 min after their application. After 90 and 300 min, the hydration and TEWL measurements were taken. The final result was the arithmetic mean (from each volunteer) of 5 independent measurements (skin hydration) and 20 measurements (TEWL). The change in skin hydration and the change in TEWL were calculated by the formulas:

$$\%\;\Delta \;\mathrm{Skin}\;\mathrm{moisture}=\frac{M1-M0}{M0}\;\times \;100\%$$

$$\%\;\Delta \;\mathrm{TEWL}=\frac{\mathrm{TEWL}1-\mathrm{TEWL}0}{\mathrm{TEWL}0}\;\times \;100\%$$

where M1 (TEWL1)—mean skin hydration (TEWL) after t (90 or 300 min) time for the test field. M0 (TEWL0—mean skin hydration (TEWL) after t (90 or 300 min) time for the control field.

### 3.9. Statistical Analysis

Values of different parameters were expressed as the mean ± standard deviation (SD). The two-way analysis of variance (ANOVA) and Bonferroni posttest between groups were performed at the level *p* value of <0.05 to evaluate the significance of differences between values. Statistical analyses were performed using GraphPad Prism 8.4.3 (GraphPad Software, Inc., San Diego, CA, USA) and Statistica 9.0 (StatSoft, CA, USA) using One-way ANOVA and Tukey’s test.

## 4. Conclusions

As part of this work, the unusual properties of extracts obtained from *Cannabis sativa* L. were shown. Comparing both extraction methods, it can be concluded that the UAE is a more efficient method, leading to obtaining more organic compounds in the tested plant. The analysis confirmed the ultrasonic extraction resulted in a higher concentration of cannabinoids, phenolic compounds, flavonoids and chlorophyll in extracts of *Cannabis sativa* L. In addition to the previously known antioxidant properties of the tested extracts, which can have a positive effect on the structure and condition of skin cells, this work also shows other benefits of hemp extracts. Due to the constantly growing popularity of hydrogel preparations in cosmetology and dermatology, the results presented in this work may contribute to the development of new hydrogels containing hemp extracts or individual compounds isolated from *Cannabis sativa* L. herb. The abilities of inhibiting matrix metalloproteinases, collagenase and elastase, presented for the first time in this work, as well as proven antioxidant properties make these extracts valuable ingredients for the production of a wide range of products that can be used in the treatment and care of the skin. Due to the high demand for preparations that inhibit the aging processes of the skin, the effect of hemp extracts on skin hydration and the possibility of preventing the degradation of collagen and elastin fibers presented here indicates the value of these extracts. The lack of a negative effect on the metabolic activity and viability of skin cells indicate the legitimacy of including hemp extracts in the recipes of skin care cosmetics as well as medicinal preparations. However, further research is obviously needed to determine the detailed mechanisms of action of these extracts, however the results presented in this paper seem to be promising.

## Figures and Tables

**Table 1:** Total content of polyphenols (TPC), flavonoids (TFC) and chlorophyll in ultrasound assisted (UAE) and magnetic stirrer assisted (MAE) hemp extracts (DWE—dry weight of extract, DWH—dry weight of herb). The content of phenolic compounds was determined using gallic acid (GAE) and flavonoids using quercetin (QE).

	TPC [mg GAE/g DWE]	TFC [mg QE/g DWE]	Chlorophyll a [mg/g DWE]	Chlorophyll b [mg/g DWE]	Chlorophyll a + b [mg/g DWE]
**MAE**	42.524 ± 0.005 ^a^	8.091 ± 0.010 ^a^	1.923 ± 0.04 ^a^	0.241 ± 0.015 ^a^	2.642 ± 0.023 ^a^
**UAE**	51.322 ± 0.012 ^b^	10.374 ± 0.009 ^b^	4.372 ± 0.022 ^b^	0.821 ± 0.010 ^b^	5.404 ± 0.042 ^b^
	**TPC** **[mg GAE/g DWH]**	**TFC** **[mg QE/g DWH]**	**Chlorophyll a [mg/g DWH]**	**Chlorophyll b [mg/g DWH]**	**Chlorophyll a + b [mg/g DWH]**
**MAE**	2.511 ± 0.011 ^a^	0.483 ± 0.011 ^a^	0.113 ± 0.005 ^a^	0.014 ± 0.016 ^a^	0.156 ± 0.019 ^a^
**UAE**	3.184 ± 0.008 ^b^	0.642 ± 0.005 ^b^	0.271 ± 0.009 ^b^	0.051 ± 0.015 ^b^	0.335 ± 0.022 ^b^

^a,b^ Different letters in the table indicate significant differences between groups (*p* < 0.05).

**Table 2:** The content of individual cannabinoids in hemp UAE and MAE extracts (DW—dry weight of extract).

Chemical Compound	MAE [mg/g DW]	UAE [mg/g DW]
Cannabidiol (CBD)	12.00 ± 1.43	31.00 ± 2.86
Cannabidiol acid (CBD-A)	130.00 ± 1.92	150.00 ± 16.84
Cannabigerol (CBG)	Not detected	Not detected
Cannabigerolic acid (CBG-A)	4.20 ± 0.38	6.30 ± 0.52
Delta-9-tetrahydrocannabinol (THC)	2.60 ± 0.19	4.00 ± 0.34
Tetrahydrocannabinolic acid (THC-A)	4.10 ± 0.46	6.50 ± 0.52
Cannabinol (CBN)	Not detected	Not detected

## Data Availability

Data is contained within the manuscript.

## Sample Availability

Samples of the compounds used and the plants extracts tested are available from the authors.
